# Mitochondrial Ca^2+^ uptake controls actin cytoskeleton dynamics during cell migration

**DOI:** 10.1038/srep36570

**Published:** 2016-11-09

**Authors:** Julien Prudent, Nikolay Popgeorgiev, Rudy Gadet, Mathieu Deygas, Ruth Rimokh, Germain Gillet

**Affiliations:** 1 Medical Research Council, Mitochondrial Biology Unit, Cambridge Biomedical Campus, Hills Road, Cambridge CB2 0XY, UK; 2 Université de Lyon, Centre de recherche en cancérologie de Lyon, U1052 INSERM, UMR CNRS 5286, Université Lyon I, Centre Léon Bérard, 28 rue Laennec, 69008 Lyon, France; 3 Hospices civils de Lyon, Laboratoire d’anatomie et cytologie pathologiques, Centre Hospitalier Lyon Sud, Chemin du Grand Revoyet, 69495 Pierre Bénite, France

## Abstract

Intracellular Ca^2+^ signaling regulates cell migration by acting on cytoskeleton architecture, cell directionality and focal adhesions dynamics. In migrating cells, cytosolic Ca^2+^ pool and Ca^2+^ pulses are described as key components of these effects. Whereas the role of the mitochondrial calcium homeostasis and the Mitochondria Cacium Uniporter (MCU) in cell migration were recently highlighted *in vivo* using the zebrafish model, their implication in actin cystokeleton dynamics and cell migration in mammals is not totally characterized. Here, we show that *mcu* silencing in two human cell lines compromises their migration capacities. This phenotype is characterized by actin cytoskeleton stiffness, a cell polarization loss and an impairment of the focal adhesion proteins dynamics. At the molecular level, these effects appear to be mediated by the reduction of the ER and cytosolic Ca^2+^ pools, which leads to a decrease in Rho-GTPases, RhoA and Rac1, and Ca^2+^-dependent Calpain activites, but seem to be independent of intracellular ATP levels. Together, this study highlights the fundamental and evolutionary conserved role of the mitochondrial Ca^2+^ homeostasis in cytoskeleton dynamics and cell migration.

Cell migration contributes to a number of physiological processes including embryonic development, wound healing and immune response. Abnormal cell migration is often associated with cancer progression and invasion^1^. Cell migration is regulated by external signals and internal factors, including actin cytoskeleton remodeling and regulation of the focal adhesion proteins (FAPs), which participate in key interactions with the extracellular matrix and the cytoskeleton^2^,^3^. Intracellular forces generated by FAPs allow the rear-to-front retraction and assembly of actin protrusions, allowing the cell to move^4^. The turnover of FAPs is spatiotemporally finely controlled by intracellular Ca^2+^ signaling. Indeed, cell retraction is regulated by the Rho GTPases-dependent actomyosin contraction^5^,^6^ and FAPs disassembly^7^,^8^, both processes being Ca^2+^-dependent. Actomyosin contraction is controlled by the phosphorylation of Myosin-Light Chain (MLC) by the Ca^2+^-Calmodulin MLC kinase pathway^9^,^10^, whereas the Ca^2+^-dependent proteases Calpains are involved in FAPs disassembly^7^,^11^,^12^.

Mitochondria have a central role in the control of the intracellular Ca^2+^ levels and signaling; they constantly uptake Ca^2+^ ions under physiological conditions, to ensure their proper functions^13^. These organelles can rapidly uptake substantial amounts of Ca^2+^ though the existence of Ca^2+^ hot spots localized at the interface between the mitochondria and the endoplasmic reticulum (ER)^14^. The mitochondrial Ca^2+^ uptake capacities have been also linked to an efficient Store-Operated Ca^2+^ Entry (SOCE)^15^,^16^,^17^,^18^. Interestingly, the role of the SOCE process, which is regulated in part by the ER-resident Stromal Interacting Molecule 1 (STIM1) and Calcium release-activated calcium channel protein 1 (Orai1), has been highlighted in the actomyosin contractility^19^,^20^ and breast tumor cell migration^21^.

Recent characterization of the mitochondrial Ca^2+^ uptake machinery, including the mitochondrial Ca^2+^ uniporter (MCU)^22^,^23^ and associated regulators^24^,^25^,^26^, shed new light on the molecular mechanisms underlying mitochondrial Ca^2+^ buffering and homeostasis. Although the phenotype of the MCU knock-out (KO) mice is mild^27^, tissue-specific KOs^28^,^29^,^30^,^31^ as well as genetic manipulations of *mcu* in other animal models^32^,^33^,^34^ provided evidence for different physiological functions of MCU^35^. Using zebrafish as a model, we recently demonstrated that MCU is involved in the control of the first embryonic cell movements^32^. Indeed *mcu* silencing led to profound migration defects in the pluripotent stem cells, thus altering anteroposterior axis formation. Subsequent studies on MCU and MICU1 in mammalian cells showed an evolutionarily conserved contribution of the mitochondrial Ca^2+^ uptake machinery in cell migration. Indeed, in endothelial^36^ or breast^37^ and cervical cancer^38^ cells, alteration of the mitochondrial Ca^2+^ uptake led to similar defects in migration abilities. Finally, computed data from clinical studies suggested that *mcu*-overexpression and *micu1*-under expression were associated with poor prognosis for breast cancer patients^38^,^39^. Despite these observations, the downstream molecular actors of this process remain unknown.

Here, we show that silencing *mcu* gene expression in human breast cancer and HeLa cells led to an actin cytoskeleton stiffness, loss of cell polarity as well as impairment of focal adhesion dynamics. Indeed, the efficient assembly/disassembly of FAPs, including Vinculin and Paxillin, was found to rely on intact mitochondrial Ca^2+^ uptake. At the molecular level, the effect of *mcu* silencing appeared to be mediated by a significant decrease of Rho-family GTPases and Calpain activities, as a result of the global decrease of cytosolic and ER Ca^2+^ pools. Together, our results support a new role of the mitochondrial Ca^2+^ homeostasis in cytoskeleton dynamics and cell migration.

## Results

### Mitochondrial Ca^2+^ uptake is required for efficient cell migration

**Figure 1:**
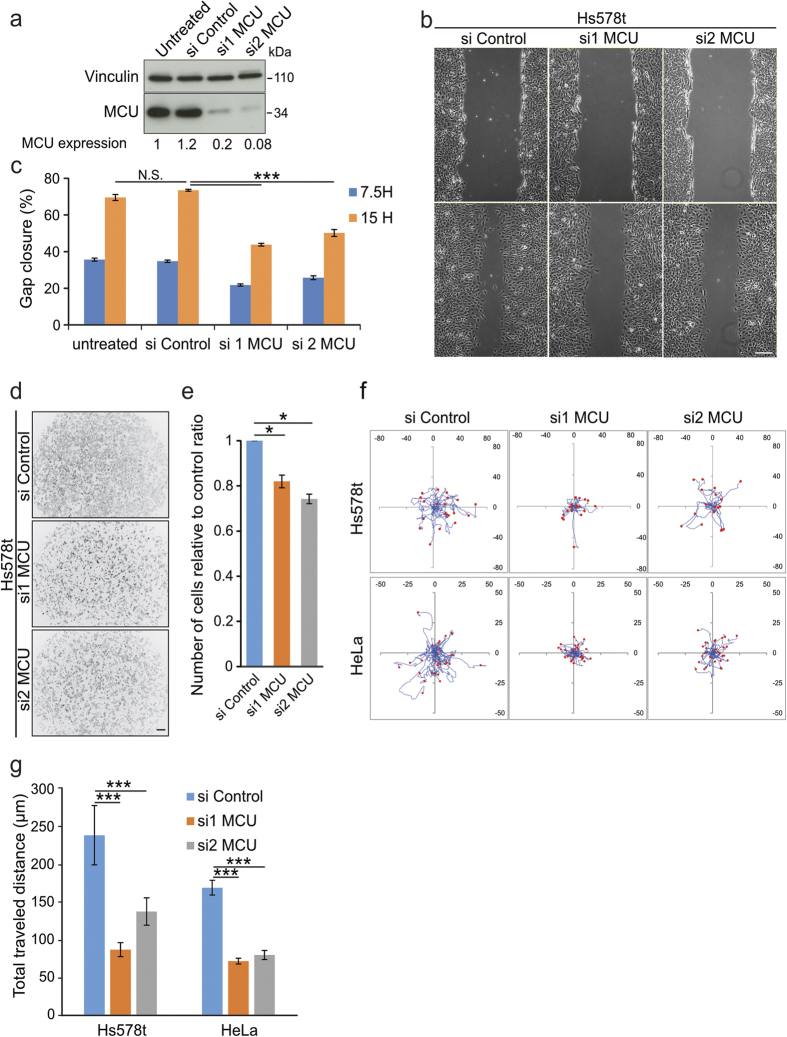
MCU knockdown induces cell migration defects. (**a)** Immunoblot blot showing the decrease of MCU protein level in Hs578t cells transfected with two siRNAs (si1 and si2) targeting *mcu* transcript. Scrambled siRNA (si Control) was used as negative control. Vinculin was used as loading control. Quantitative ratios (MCU expression) between MCU and Vinculin were indicated below. (**b)** Representative images of the effect of *mcu*-silencing on Hs578t migration performed by wound healing assay. Compared to control cells, *mcu*-silenced cells present decreased capacities to close the gap. Scale bar: 200 μm. (**c**) Histogram representing the percentage of gap closure estimated at 7.5 and 15 hours post-wound from (**b**) (mean ± S.E.M; n = 4 independent experiments). N.S. = not significant; ***P < 0.001. **(d)** Representative Grayscale images of the effect of *mcu*-knockdown on Hs578t invasion performed with Boyden chamber assay using Fetal Bovine Serum (FBS) as chemoattractant. Compared to controls, less *mcu*-silenced cells have crossed the membrane. Scale bar: 200 μm. (**e)** Histogram representing the number of invading cells relative to control condition measured at 7.5 hours post-seeding from (**d**) (mean ± S.E.M; n = 3 independent experiments). *P < 0.05. (**f)** Representative single cell tracking experiments highlighting the cell paths (blue lines) of isolated Hs578t (red dots) or HeLa cells (red dots), silenced or not for *mcu*; acquisition time 24 hours. Compared to control cells, *mcu*-silenced cells have shorter migration paths. (**g)** Histogram deduced from the results displayed in (**f**) representing the distance of cell migration relative to control (mean ± S.E.M; 30 cells in each experiment; n = 3 independent experiments). ***P < 0.001. See also Supplementary Fig. S1.

To investigate the role of the mitochondrial Ca^2+^ uptake in cell migration, we analyzed the effect of *mcu* silencing. Two specific short interfering RNAs (siRNAs) were directed to the 3′ UTR region of the *mcu* transcript, hereafter called si1 and si2 MCU. These siRNAs efficiently decreased the levels of the endogenous MCU protein (Fig. 1a), and led to a significant decrease in the capacity of mitochondria to uptake exogenous Ca^2+^ (Supplementary Fig. S1a–d). First, in the highly migrating Hs578t breast cancer cell line, we analyzed the effect of *mcu* silencing on their capacities to close the gap in a classical wound-healing assay. As shown in Fig. 1b,c, *mcu* knockdown reduced significantly the ability of Hs578t cells to close the wound (43.8% ± 0.7%; 50% ± 1.8% of gap closure for si1 and si2 MCU at 15 hours post wound, respectively) compared to control cells (73.5% ± 0.5% of gap closure). We next examined the capacity of Hs578t cells to migrate through a basement membrane following a serum gradient using a Boyden chamber assay. Compared to control cells, a significantly lower percentage of *mcu-*silenced cells were found to have crossed the membrane after 7.5 hours (81.9% ± 2.8%; n = 9557 cells counted and 74.2% ± 2.1%; n = 8752 cells counted, for si1 and si2 MCU respectively, compared to 100% for control cells; n = 11728 cells counted) (Fig. 1d,e).

Finally, we examined the effect of *mcu*-knockdown by directly tracking individual cells and measuring the total traveled distance (TTD) over a 24-hours period. *Mcu* knockdown in Hs578t significantly reduced cell migration paths compared to controls. Whereas in control cells TTD reached 240.1 μm ± 38.5 μm (n = 60 cells), *mcu*-silenced cells showed a decreased migration rate, TTD being lowered to 87.7 μm ± 9.3 μm and 138.5 μm ± 18.3 μm (n = 60 cells) for si1 and si2 MCU Hs578t cells, respectively (Fig. 1f,g). Of note, these results were confirmed in a second cell line, HeLa cells, with lower migration rate (Fig. 1f,g). Interestingly, cells treatment with Ru360, a potent MCU inhibitor, led to a similar cell migration defect, highlighting the specific role of the mitochondrial Ca^2+^ uptake during cell migration (Supplementary Fig. S1e).

Importantly, the cell migration phenotype was not found to be associated with cell proliferation or apoptotic program defects. Indeed, *mcu*-silenced Hs578t cells did not show any decrease in cell proliferation or increase in apoptosis (Supplementary Fig. S1f,g). Together, these results support the notion that mitochondrial Ca^2+^ uptake is required for optimal migration of human cells.

### MCU downregulation leads to impaired cell polarization and Rho GTPases activities

**Figure 2:**
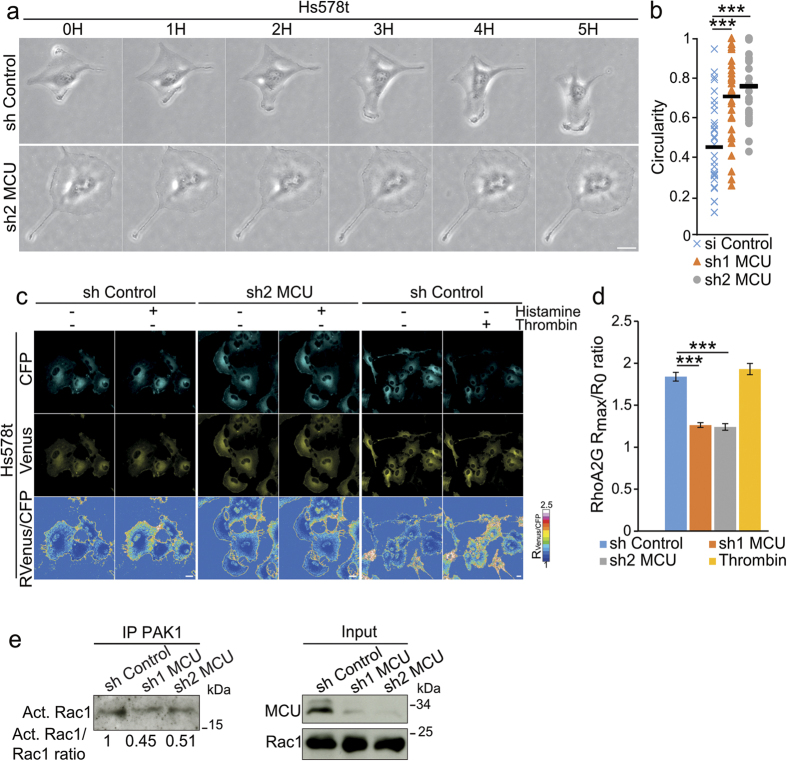
Mitochondrial Ca^2+^ uptake impairment leads to cell polarization defects and decreased small GTPases activities. (**a)** Representative transmission images of single cell tracking of Hs578t cells stably expressing shRNA control versus shRNA targeting *mcu* transcript. Cells expressing the control shRNA have forward-to-rear polarization, which was absent in shMCU cells. Scale bar: 20 μm. (**b**) Graph representing the quantification of circularity coefficients for sh Control (n = 34 cells), sh1 MCU (n = 32 cells) and sh2 MCU (n = 29 cells) (mean ± S.E.M; three independent experiments). Compared to control cells, shMCU cells present an increased circularity coefficient. ***P < 0.001. (**c)** Confocal time-lapse images showing the CFP (cyan), Venus (yellow) as well as Venus/CFP ratio (R_Venus/CFP_) of the RhoA2G biosensor dye following IP_3_R mobilization using Histamine 100 μM in Hs578t cells. Thrombin was used as positive control for RhoA activation. Ratio was visualized as a heat map using false colors. Images were acquired before and after 1 min treatment with Histamine or Thrombin. Following Histamine treatment, RhoA activation is higher in control cells compared to shMCU cells. Scale bar: 20 μm. (**d**) Histogram showing the R_max_/R_0_ ratio following Histamine or Thrombin stimulations in control versus *mcu*-silenced cells, from (**c**). (mean ± S.E.M.; 20 cells in each experiment; n = 3 independent experiments). **P < 0.01, ***P < 0.001. (**e**) Immunoblot showing the deacread Rac1 activation (Act. Rac1) in sh MCU compared to sh control cells. Activated Rac1 in Hs578t cells was purified using PAK1 beads and detected using specific Rac1 antibody. Total Rac1 levels were used to normalize the levels of activated Rac1 (numbers below). See also Supplementary Fig. S2.

To further characterize the effects of mitochondrial Ca^2+^ uptake reduction on cell migration we analyzed the behavior of individual cells lacking MCU using time-lapse videomicroscopy. To perform these experiments we established stable Hs578t MCU negative cells by infection with lentiviruses encoding short hairpin RNAs (shRNAs) targeting the same regions as si1 and si2 MCU siRNAs. ShMCU expressing clones presented the same migration impairment (Supplementary Fig. S2a,b) and decreased mitochondrial Ca^2+^ uptake (Supplementary Fig. S2c,d), as siRNAs transfected cells. Cells expressing a control shRNA exhibited, in a coordinated fashion, lamellipodia formation at the leading edge and disassembly of adhesion points at the rear end of the cell body, allowing them to migrate (Fig. 2a). These cells presented a typical forward-to-rear polarization, which was absent in shMCU cells. These morphological changes were quantified by measuring the circularity coefficient of the cells (from 0, to an infinitely elongated polygon, to 1 for a perfect circle). Compared to controls, which showed a circularity coefficient of 0.44 ± 0.21 (n = 34 cells), *mcu* knockdown cells showed an increased circularity coefficient (0.70 ± 0.22; n = 32 cells and 0.75 ± 0.16; n = 29 cells, for sh1 and sh2 MCU cells, respectively), indicating a more circular shape (Fig. 2b).

Lamellipodia dynamics is under the control of the Rho family of GTPases, including Rac1 and RhoA, which are essential for the formation and retraction of lamellipodia^6^. We first analyzed the activity of RhoA using Fluorescence Resonance Energy Transfer (FRET)-based biosensor RhoA2G^40^. RhoA2G activity was calibrated using thrombin, which has previously been shown to activate RhoA^41^. To measure Ca^2+^ related RhoA activation we used histamine to induce IP_3_-dependent Ca^2+^ release from the ER. In these conditions the amount of activated RhoA was significantly decreased in both shMCU Hs578t clones (RhoA2G R_max_/R_0_ ratio = 1.26 ± 0.24 and 1.24 ± 0.28 for sh1 and sh2 MCU, respectively; n = 20 cells per condition) compared to the control (RhoA2G R_max_/R_0_ ratio = 1.84 ± 0.41, n = 20 cells) (Fig. 2c,d). In addition we measured basal Rac1 activation using PAK1-based immunoprecipitation assay. Compared to control cells, sh1 and sh2 MCU clones showed decreased levels of activated Rac1 (Fig. 2e), suggesting that mitochondrial Ca^2+^ uptake is important for Rac1 and RhoA activation.

### Mitochondrial Ca^2+^ uptake impairment induces cytoskeleton stiffness and focal adhesion dynamics defects

**Figure 3:**
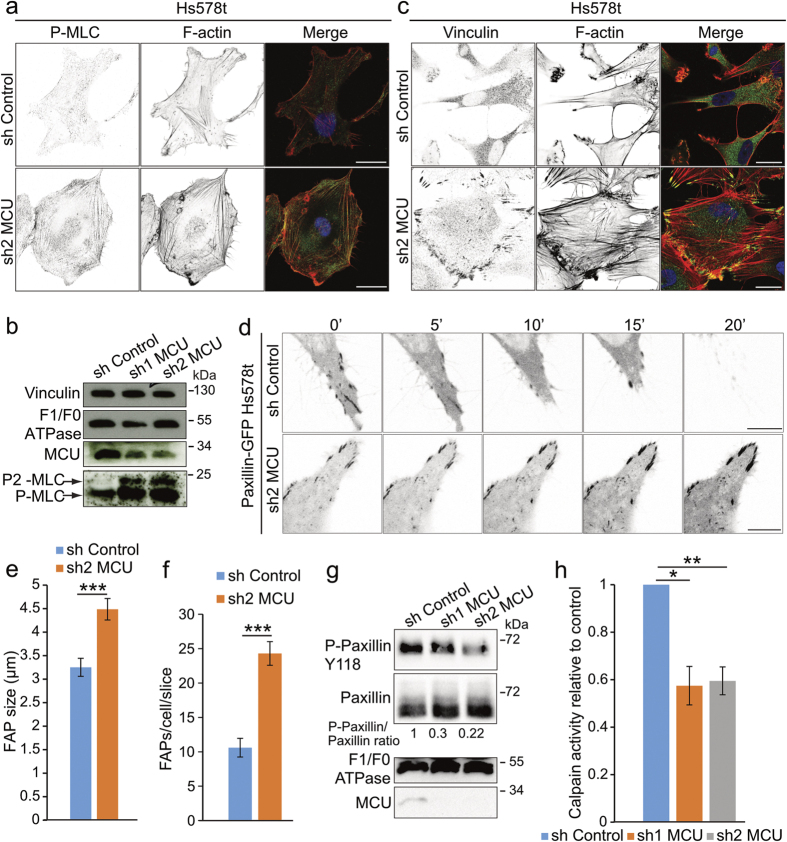
Mitochondrial Ca^2+^ uptake is required for cytoskeleton and focal adhesion protein dynamics. (**a**) Representative confocal images showing the accumulation of phospho-myosin light chain (P-MLC) signal in F-actin fibers in sh2 MCU cells compared to controls. P-MLC and F-actin were stained using P-MLC antibody and phalloidine rhodamine probe, respectively. Merge channels between P-MLC (green) and F-actin (red) were presented in the rightmost panels. Scale bar: 20 μm. (**b**) Immunoblot showing the increase of MLC phosphorylation on Serine19 (P-MLC) and Threonine18 and Serine19 (P_2_-MLC) positions in *mcu*-silenced cells. MCU antibody was used to show the efficiency of the shRNAs. F_1_F_0_ ATPase antibody was used as loading control. (**c**) Representative confocal images showing the accumulation of Vinculin in FAPs in sh2 MCU cells compared to control. Vinculin and F-actin were detected using anti-Vinculin antibody and phalloidine rhodamine probe, respectively. Merge channels between Vinculin (green) and F-actin (red) were presented in the rightmost panels. Scale bar: 20 μm. (**d**) Representative time-lapse confocal images showing the persistence of Paxillin-GFP signal in the FAPs of *mcu*-knockdown cells compared to control. Scale bar: 5 μm. (**e**) Histogram representing the increase of FAPs number estimated from Vinculin staining in sh2 MCU cells compared to control. (mean ± S.E.M; 10 cells for each condition; n = 3 independent experiments). ***P < 0.001. (**f**) Histogram representing the increase of FAP size estimated from Vinculin staining in sh2 MCU cells compared to control cells. (mean ± S.E.M; 10 cells for each condition; n = 3 independent experiments). ***P < 0.001. (**g**) Immunoblot showing the effect of sh1 and sh2 MCU on phospho-Paxillin Y118 protein levels (P-Paxillin Y118). Quantitative ratios between P-Paxillin Y118 and total Paxillin are indicated below. *Mcu* knockdown leads to decreased P-Paxillin Y118/total Paxillin ratio. MCU antibody was used to show the efficiency of the shRNAs. F_1_F_0_ ATPase antibody was used as loading control. (**h**) Histogram presenting the decrease of Calpain activity measured in *mcu*-silenced cells and compared to control cells (mean ± S.E.M; n = 3 independent experiments). *P < 0.05, **P < 0.01. See also Supplementary Fig. S3.

The involvement of Rho GTPases in cell polarity and migration is essentially driven by their control of cytoskeleton organization and dynamics^6^. We thus analyzed the three major cytoskeleton structures: microtubules, intermediate filaments and microfilaments. The overall organization of the microtubules and intermediate filaments using alpha-Tubulin and Vimentin as markers, respectively, showed no significant difference in *mcu-*silenced cells, compared to controls (Supplementary Fig. S3). However, profound alterations of central microfilament components, including Myosin-Light Chain (MLC) phosphorylation and F-actin fibers, were detected in the cells lacking MCU. Compared to control cells, *mcu*-silenced cells presented increased phosphorylation levels of MLC residues Thr18 and Ser19, as shown by immunofluorescence (Fig. 3a) and western blotting (Fig. 3b). The observed cytoskeleton stiffness was correlated to a higher density of the F-actin fibers and actin bundles as detected by phalloidin-rhodamine staining (Fig. 3a,c). In fact, actin bundles attach to extracellular matrix through focal adhesion points (FAPs) to provide the mechanical forces required for adhesion, retraction and efficient migration^42^. We thus checked the status of FAPs by analyzing the distribution of the Vinculin protein. As shown in Fig. 3c, in shMCU cells, Vinculin mainly accumulated in FAPs, in contrast to control cells where Vinculin localization was more diffuse. In fact, the changes of Vinculin localization in *mcu*-silenced cells were not linked to a variation in Vinculin protein levels (Fig. 3b), suggesting that it could be rather due to alterations in the dynamics of FAPs formation and disassembly. To test this hypothesis we analyzed FAPs dynamics by monitoring Paxillin-GFP expressing cells with fluorescent time-lapse confocal microscopy. In control cells, Paxillin-GFP staining gradually decreased at the tailing edge, allowing the cells to move forward. However, in shMCU cells, Paxillin-GFP staining remained persistent and became even higher during the 20-min time period of monitoring (Fig. 3d). These observations suggested that the FAPs dynamic and structure in *mcu-*silenced cells were impaired. Quantification of the number of FAPs per cell as well as size measurement revealed a significant increase of both parameters in *mcu-*silenced cells (Fig. 3e,f). We further monitored the Paxillin phosphorylation level at Tyr 118. Paxillin phosphorylation is orchestrated by the focal adhesion kinase (FAK) and associated with the assembly and turnover of the adhesions^43^. Moreover, Phospho-Paxillin provides a docking site for recruitment of other signaling molecules required for cell migration. Interestingly, Paxillin phosphorylation was strongly decreased in shMCU cells, as shown by the Phospho-Paxillin/Total Paxillin ratio (Fig. 3g), corroborating the observed Paxillin-GFP dynamics in live cell imaging. Paxillin turnover in FAPs is controlled by the Ca^2+^-dependent, non-lysosomal cysteine proteases known as Calpains. We therefore analyzed the effet of *mcu* silencing on global Calpains activity. As shown in Fig. 3h, Calpains activity showed a 40% decrease in *mcu-*silenced cells (57.4% ± 18.1% and 59.5% ± 11.7% for sh1 and sh2 MCU respectively, compared to 100% for control cells). Together, these results suggest that mitochondrial Ca^2+^ uptake, acts on cell migration by controlling F-actin microfilaments and focal adhesion dynamics, in a Calpain-dependent manner.

### MCU downregulation leads to reduced intracellular Ca^2+^ levels

**Figure 4:**
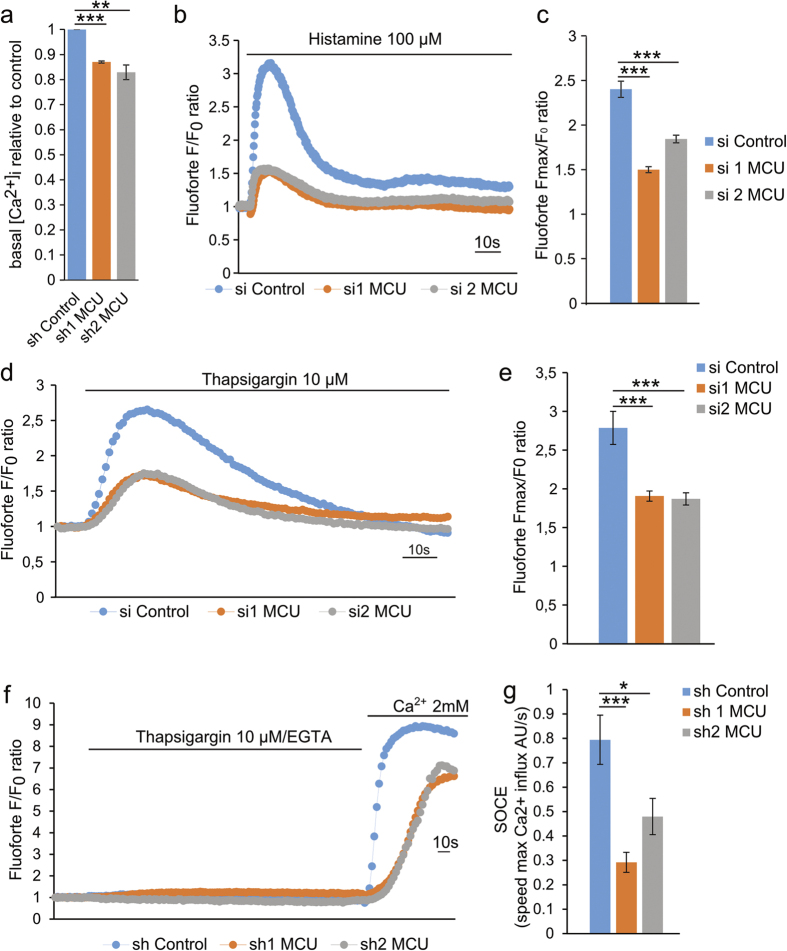
MCU knockdown results in intracellular Ca^2+^ impairment. (**a)** Histogram showing the basal cytosolic Ca^2+^ levels ([Ca^2+^]_i_) of sh Control and sh1, sh2 MCU Hs578t cells using the cytosolic Ca^2+^ sensitive dye Fluoforte. Compared to control cells, *mcu*-silenced cells have lower basal cytosolic [Ca^2+^]_i_. Fluorescence intensities were reported as ratio to sh Control cells (mean ± S.E.M; n = 3 independent experiments; **P < 0.01, ***P < 0.001. (**b**) Representative trace of cytosolic Ca^2+^ rise following histamine (100 μM) stimulation using Fluoforte dye performed in control or *mcu*-silenced cells. *Mcu*-silencing led to a decrease in the ER-dependent cytosolic Ca^2+^ rise. Fluoforte fluorescence intensities were normalized to the baseline (F/F_0_ ratio). (**c)** Histogram depicting the maximum Ca^2+^-increase in the cytosol (F_max_/F_0_ ratio) in cells following histamine (100 μM) treatment, from (**b**). (mean ± S.E.M; n = 3 independent experiments). ***P < 0.001. (**d**) Representative trace of cytosolic Ca^2+^ rise following ER-Ca^2+^ leak induced by Thapsigargin (10 μM) stimulation. *Mcu*-silencing led to a decrease in the ER-dependent cytosolic Ca^2+^ rise. Fluoforte fluorescence intensities were normalized to the baseline (F/F_0_ ratio). (**e)** Histogram depicting the maximum Ca^2+^-release in the cytosol (F_max_/F_0_ ratio) in cells following thapsigargin (10 μM) treatment, from (**d**). (mean ± S.E.M; n = 3 independent experiments). ***P < 0.001. (**f)** Representative trace of the relative changes in the Fluoforte fluorescence intensities normalized to the baseline (F/F_0_ ratio). Following Thapsigargin (10 μM)-induced ER Ca^2+^ depletion, extracellular Ca^2+^ (2mM) was applied on the cells in order to measure SOCE efficiency corresponding to the maximum speed of Ca^2+^ influx. *Mcu*-silencing led to decrease SOCE efficiency compared to control cells. (**g)** Histogram depicting SOCE efficiency (speed max Ca^2+^ influx) measured in Hs578t cells stably expressing sh Control, sh1 MCU or sh2 MCU shRNAs, from (**f**) (mean ± S.E.M; n = 3 independent experiments) *P < 0.05; ***P < 0.001. See also Supplementary Fig. S4.

Calpains are intracellular proteases, which require cytosolic Ca^2+^ ions for their activity^44^. We thus analyzed the basal cytosolic Ca^2+^ level in control versus *mcu*-silenced Hs578t cells, using the cytosolic Ca^2+^ dye Fluoforte. As shown in Fig. 4a, *mcu* silencing led to a significant decrease in the basal cytosolic Ca^2+^ pool (decrease of 13% and 17.1% for sh1 and sh2 MCU cells, respectively). These cytosolic and mitochondrial Ca^2+^ uptake reductions did not appear to correlate with a decrease in the mitochondrial bioenergetics. Indeed basal oxygen consumption rates (OCR) in *mcu*-silenced cells were found to be similar to control cells (Supplementary Fig. S4a,b). In the same line, we checked the global intracellular ATP levels in *mcu*-silenced and control cells. Actually, as shown in Fig. S4c, the global intracellular ATP levels were similar in *mcu-silenced* cells (6.22 μmol/g ± 0.1 and 7.65 μmol/g ± 0.25 for shRNA MCU #1 and #2, respectively), compared to controls (6.81 μmol/g ± 0.31). Together, these results indicate that the observed F-actin cytoskeleton phenotype is not due to a global intracellular ATP production default.

The decrease of basal intracellular Ca^2+^ levels in *mcu*-silenced cells suggested that intracellular compartments, including the ER, may have reduced Ca^2+^ buffering capacities. To test this hypothesis, we measured IP_3_-dependent ER Ca^2+^ release following histamine treatment. In control cells, histamine induces a rapid increase of cytosolic Ca^2+^ levels (2.4 fold; ± 0.09), in contrast to *mcu-*silenced cells (1.5 fold; ±0.03 for sh1 MCU; 1.8 fold; ±0.04 for sh2 MCU) (Fig. 4b,c).

The ER Ca^2+^ pool is directly dependent on the store operated Ca^2+^ entry (SOCE), which refills the ER lumen with Ca^2+^ ions from extracellular space^45^. This led us to analyze the passive ER Ca^2+^ leakage, by inhibiting the ER Ca^2+^ pump sarcoplasmic-endoplasmic reticulum Ca^2+^ ATPase (SERCA) with thapsigargin. In control cells, thapsigargin treatment induces a progressive increase of cytosolic Ca^2+^ levels, peaking at about 4 min post-incubation (Fluoforte F_max_/F_0_ ratio  = 2.79 ± 0.67) (Fig. 4d,e). In *mcu-*silenced cells, the ER Ca^2+^ leak was significantly decreased (Fluoforte F_max_/F_0_ ratio = 1.90 ± 0.21 and 1.87 ± 0.29 for si1 and si2 MCU, respectively) indicating a decrease of ER Ca^2+^ levels (Fig. 4d,e). Finally, we directly measured SOCE efficiency by inducing ER Ca^2+^ leakage by thapsigargin treatment in a Ca^2+^ free medium, followed by extracellular Ca^2+^ boost. As shown in Fig. 4f,g, in *mcu*-silenced cells, SOCE activity was strongly reduced (intracellular Ca^2+^ influx = 0.29 AU/s ± 0.04 AU/s for sh1 MCU; 0.48 AU/s ± 0.07 AU/s for sh2 MCU) compared to control cells (0.8 AU/s ± 0.1 AU/s). This was further confirmed using ionomycine to deplet the ER Ca^2+^ stores (Supplementary Fig. S4d,e). Altogether these results support the notion that the down regulation of the mitochondrial Ca^2+^ uptake in *mcu-*silenced cell, leads to a decrease of ER and cytosolic Ca^2+^ pools due to impairment of SOCE.

### Cytosolic Ca^2+^ depletion phenocopies *mcu* knockdown phenotype

To confirm that direct SOCE impairment leads to cell migration and cytoskeleton defects similar to those observed following MCU silencing, we silenced STIM1 using three specific siRNAs (Supplementary Fig. S5a). In fact, all three siRNAs led to a significant decrease of cell migration (TTD of 160.70 μm ± 11.6 μm, 202.5 ± 16.6 μm, 185.50 μm ± 12.9 μm, compared to 383.7 μm ± 19.6 μm for si1–3 STIM1 and si Control, respectively) (Supplementary Fig. S5b,c) together with Vinculin accumulation in the FAPs (Supplementary Fig. S5d). These results show that the SOCE-dependent Ca^2+^ uptake sustains the migration capacities of Hs578t breast cancer cells.

**Figure 5:**
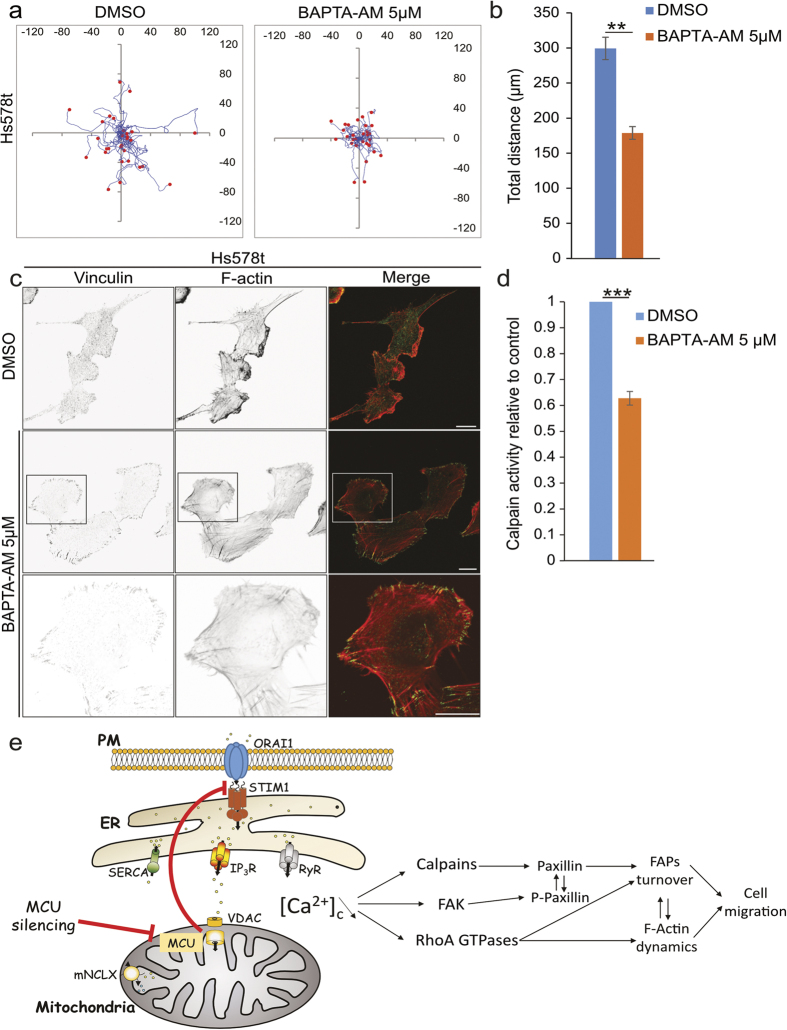
Pharmacological impairment of intracellular Ca^2+^ levels phenocopies *mcu* silencing. (**a)** Representative cell paths (blue lines) of isolated Hs578t cells (red dots) treated with DMSO or the cell permeable Ca^2+^ chelator BAPTA-AM (5 μM); acquisition time 24 hours. BAPTA-AM led to decrease cell migration. (**b)** Histogram showing the decrease in the cell migration of BAPTA-AM treated cells (mean ± S.E.M; 30 cells in each experiment; n = 3 independent experiments). **P < 0.01. **(c)** Representative confocal images showing the accumulation of Vinculin in the FAPs in BAPTA-AM treated cells compared to DMSO treated controls. Vinculin and F-actin were stained using anti-Vinculin antibody and phalloidine rhodamine probe, respectively. Scale bar: 20 μm. (**d)** Histogram representing the Calpain activity measured in BAPTA-AM treated cells reported to control cells (mean ± S.E.M; n = 3 independent experiments). BAPTA-AM treatment led to a decrease Calpain activity. ***P < 0.001. (**e)** Schematic model showing the role of MCU in the control of Ca^2+^ homeostasis and cell migration. *Mcu*-silencing using si or shRNA strategies leads to SOCE impairment with subsequent decrease of the cytosolic Ca^2+^ levels ([Ca^2+^]_c_). This intracellular calcium reduction causes a decrease in the Rho family of GTPases and Calpains activities, which may alter the cytoskeleton dynamics and the migration of the cell. See also Supplementary Fig. S5.

To confirm the actual importance of cytosolic Ca^2+^, we treated Hs578t cells with the cell permeable Ca^2+^ chelator BAPTA-AM. Incubation with 5 μM of BAPTA-AM significantly decreased cell migration as measured by individual cell tracking over a period of 24 hours (Fig. 5a). Indeed, in BAPTA-AM treated cells the TTD was 178.8 μm ± 8.9 μm, compared to 299.4 μm ± 15.4 μm for control cells (Fig. 5b). Furthermore, BAPTA-AM treatment resulted in Vinculin accumulation in the FAPs at the periphery of the cells (Fig. 5c). Finally, we detected a significant decrease in Calpains activity in BAPTA-AM treated cells (Fig. 5d). Altogether these results show that the depletion of cytosolic Ca^2+^ phenocopies at the molecular level the silencing of *mcu,* highlighting the crucial role of mitochondrial Ca^2+^ uptake in cytoskeleton architecture and cell migration (Fig. 5e).

## Discussion

Cell migration is a highly dynamic process in which actin cytoskeleton remodeling and focal adhesion turnover orchestrate front-to-rear polarity and cell movements^2^. Lamellipodia formation at the front end is controlled by Rac1, whereas actomyosin contraction at the rear end is regulated by RhoA^6^. In addition, Calpains allow the degradation of FAPs, including Paxillin, which is required for proper rear end retraction^7^. In fact, a number of these events are Ca^2+^-dependent, further supporting that Ca^2+^ trafficking is critical for cell migration. We previously showed that alterations of mitochondrial Ca^2+^ buffering due to *mcu* silencing compromises the migration of the blastomeres in the developing zebrafish^32^. Here we show that in breast cancer and HeLa cells, *mcu* silencing leads to a strong inhibition of migration and loss of polarity. At the molecular level, this seems to be due to of F-actin fibers accumulation and FAPs stabilization. This phenotype appears to be mediated by the decrease of RhoA and Rac1 activities as well as downregulation of total Calpain activity, two Ca^2+^ dependent parameters. Actually, a direct lowering of intracellular Ca^2+^ levels using BAPTA-AM or inhibition of the SOCE by silencing STIM1^18^ mimicked *mcu* knock down phenotype. Importantly, treatment of the cells with the potent MCU inhibitor Ru360, resulted in a decreased capacity of the cells to close the gap, which illustrates the primordial role of mitochondrial Ca^2+^ homeostasis in the cell migration process.

Interestingly, we also noticed a decrease of Paxillin phosphorylation on Y118, a known substrate of FAK, suggesting that this kinase might be down regulated in *mcu*-silenced cells. This hypothesis is further supported by the fact that FAK activity is regulated by the CaMKII and Calmodulin Ca^2+^-dependent pathways^46^,^47^. In addition, in these cells, the localization of FAK might also be altered since FAK association at FA^48^ and FAK/FAP interactions have been reported to depend on Ca^2+^ 49.

Given that FAK can also influence the activity of Rho-family GTPases^50^, it might be possible that the observed inhibition of small GTPases upon *mcu* silencing is due to the inhibition of FAK, thus shedding new light on the mechanisms that regulate cell migration *via* Ca^2+^ intracellular trafficking.

Cytosolic Ca^2+^ pulses are involved in cell directionality^51^ and lamellipodia retraction^8^. These microdomains, also called Ca^2+^ flickers are thought to be generated by Ca^2+^ entry from the plasma membrane^51^ and by IP_3_-induced Ca^2+^ release from the ER^20^. Here, we report that both cytosolic and ER Ca^2+^ pools are decreased in *mcu* knocked down cells, confirming the requirement of an intact mitochondrial Ca^2+^ uptake machinery to sustain SOCE^18^,^38^. In *mcu*-silenced cells, altered SOCE may thus compromise the generation of Ca^2+^ pulses, affecting in turn FAPs dynamics and cell migration. In fact, *mcu* silencing may impair the capacity of mitochondria to buffer Ca^2+^ flickers, particularly at the mitochondria/ER contact sites, where Ca^2+^ currents may take place^52^. Such mitochondrial impairment to buffer Ca^2+^ flickers may alter the physiological spatiotemporal Ca^2+^ distribution required for proper focal adhesion dynamics. This further supports the notion that the mitochondrial physiology has a role in cytoskeleton dynamics and cell migration. In this respect, we analyzed in Hs578t cells, the effect of *mcu* silencing on mitochondrial bioenergetics. Using Seahorse™ technology, we did not observe any significant difference in the OCR between control and *mcu-*silenced cells. Moreover, global intracellular ATP production measurement did not reveal any difference between *mcu-*silenced cells and controls suggesting that the observed cell migration phenotype is not due to an ATP production decrease, but is specific to the mitochondrial Ca^2+^ homeostasis deregulation.

Interestingly, it has been shown that mitochondria was involved in the ER- Ca^2+^ filling as well^53^. Indeed, in cardiac pacemaker cells under severe physiological stress, MCU is required for promoting oxidative phosphorylation and ATP production in a specific subcellular compartment required for the activity of SERCA2a and the reloading of ER Ca^2+^ stores^54^. Thus even if the global intracellular ATP production was not altered in *mcu-*silenced cells, we can not exclude that a site specific production of ATP at the mitochondria/ER contact points is specifically required for Actin cytoskeleton remodeling during cell migration.

Finally, our results, obtained in human cell lines, underscore the role of F-actin cytoskeleton dynamics as described in the nematode^34^ and the zebrafish^32^ models. Indeed, we previously showed that downregulation of MCU in zebrafish embryos led to a decrease in mitochondrial Ca^2+^ pool together with a dysregulation of F-actin protrusion dynamics, leading to major developmental defects^32^; however, the mechanism involved in actin dysregulation was not elucidated. An interesting study performed in *C. elegans* highlighted also the critical role of mitochondrial Ca^2+^ uptake in reactive oxygen species (ROS) production, which seems to be required for Actin cytoskeleton dynamics and wound healing^34^. Recently, MCU expression was linked to redox status in breast cancer cell lines. Indeed, downregulation of MCU was reported to decrease mitochondrial ROS production as well as hypoxia-inducible factor-1**α** expression, impacting cell migration and tumor progression^37^. However, in MDA-MB-213 breast carcinoma cells, manipulations of MCU and MICU1 have no effect on ROS production^39^. Further studies need to be performed to completely describe the potential connection between mitochondrial Ca^2+^ uptake, ROS generation and actin cytoskeleton dynamics.

Together, these present observations combined with previous studies reveal an additional mechanism by which mitochondrial Ca^2+^ uptake affects cytoskeleton dynamics and cell migration. Interestingly, it appears that the MCU contribution to cell migration is not restricted to cancer cells but rather reflects an evolutionary conserved process in eukaryotes, from worms to vertebrates.

## Methods

### Cell Lines

HeLa cervical cancer and Hs578t breast carcinoma cell lines, routinely checked for contaminations, were grown under standard cell culture conditions (37 °C, 5% CO_2_) in Dulbecco’s modified Eagle’s medium (DMEM) high glucose medium (Gibco) supplemented with 10% FBS (Sigma), 100 U/mL penicillin and 100 μg/mL streptomycin (Gibco).

### RNA interference

Two siRNAs duplex targeting the 3′UTR sequence of *mcu* (NM_138357.1) were selected in the dataBase of Integrated DNA technologies (IDT). The first and second siRNAs, targeting the 3′UTR sequence of *mcu* (NM_138357.1), sense strands are:

Si1 MCU: 5′-GGAUCUCAAAGGGUGCAAUUUAUCT-3′.

Si2 MCU: 5′-GGAACCAACUUAUAACUGUUUAATA-3′.

The three siRNAs sequence of *stim1* (NM_001277961.1) sense strands are:

Si1 STIM1 (targeting the CDS): 5′-GGUGCAAUAUUACAACAUCAAGAAG-3′

Si2 STIM1 (targeting the CDS): 5′-GAUGAGAUCAACCUUGCUAAGCAGG-3′

Si3 STIM1 (targeting the 3′UTR): 5′-GUUAUAAGGCAGUCACUUUUUCUCT-3′.

The Allstar negative control siRNA (Qiagen) was used as siRNA Control.

SiRNA transfection was performed using Lipofectamine RNAiMAX (Invitrogen) according to the manufacturer’s protocol. Cell analyses were performed 48 hours (h) post transfection.

### Lentiviral infection

For *mcu* stable down-regulation, two Hs578t clones were generated by lentiviral infection with pSuperRetro vector containing MCU shRNA possessing the same two sequences of the Hairpin as the siRNA. ShRNA MCU expressing cells were selected using blasticidin (5 μg/mL). RhoA activity was detected using RhoA2G FRET based biosensor encoded by pLentiRhoA2G construction (Addgene #40179). Cells expressing RhoA2G were selected with puromycin (1 μg/mL).

### Wound healing assays

Wound healing assays were performed in Hs578t cells. Briefly, 48 h post transfection cell layer at confluence was scratched in two orthogonal lines using sterile 10 μL pipette tip and washed two times with fresh medium. For Ru360 experiments (Calbiochem 557440), 4 × 10^5^ cells were plated in 6-well plates and grown overnight. Ru360 (60 μM) was added in combination with Probenecib (Molecular Probes #P36400) at 2.5 mM to avoid cellular extrusion. Pictures were taken at 0 and 7.5 h post-wounding using inverted microscope (Zeiss). Cell free space was calculated using Image J software (NIH).

### Boyden chamber migration assays

Boyden chamber migration assays were performed using FluoroBlok 96-multiwell insert plates (BD Falcon) with an 8 μm pore size PET membrane. Hs578t cells were cultured in serum-free medium 12 h prior to the cell migration assay. 1.25 × 10^5^ cells were seeded in the upper chamber in serum-free medium; medium supplemented with 5% serum was used as a chemo-attractant in the lower chamber. The plates were incubated for 7.5 h; migrating cells were stained with 2 μM Calcein-AM Fluorescent Dye (Interchim). Cells were counted using a Zeiss inverted fluorescence microscope.

### Cell tracking, proliferation and apoptosis detection

For cell tracking experiments, apoptosis and cell proliferation, Hs578t and HeLa cells were analyzed using IncuCyte live-content imaging system (Essen Bioscience). Briefly, cell tracking was performed on transfected cells seeded at 10% confluence in 24-well locked plates (Essen Bioscience). Images were automatically acquired every 30 minutes (min) for 48 h at 4X magnification (single images). Cells were tracked for 24 h; cell coordinates were acquired using ImageJ software. The total distance was calculated with Excel house made algorithm (Microsoft). Apoptosis was detected using Caspase-3/7 apoptosis assay reagent at 2.5 μM concentration (Essen Bioscience #4440). Images were automatically acquired in phase and green fluorescence channels every 30 min for 48 h at 4X magnification. Data were proceeded using a dedicated algorithm (Essen Bioscience). Cell proliferation was estimated using the phase-contrast images of the same experiments.

### Live cell imaging

Videomicroscopy experiments were performed on Hs578t, stably expressing shcontrol or sh-MCU RNAs. Cells were monitored every minute for 5 h. Pictures were taken at 20X magnification using a Zeiss Axiovert 200 microscope.

Paxillin dynamics were analyzed using Hs578t cells transiently transfected with pEGFP-Paxillin. Twenty-four h after transfection cells were seeded at 10% confluence in 8 well Nunc™ Lab-Tek™ II Chamber Slide (Thermo Scientific). Images at 60X magnification were acquired every min for 20 min for Paxillin-GFP using a Zeiss 780 confocal microscope equipped with chamber preheated at 37 °C in presence of 5% CO_2_.

### RhoA activation

RhoA activity was analyzed using Hs578t cells stably expressing RhoA2G biosensor. Twenty-four h before measurements cells were seeded at 10% confluence in 8 well Nunc™ Lab-Tek™ II Chamber Slide (Thermo Scientific). Images were acquired on isolated cells expressing low levels of the RhoA2G as previously described^40^ with the following modifications. Fluorescence excitation was performed using 405 nm laser and detection filters were set at 480/40 nm (donor channel) and 535/30 nm (FRET channel) using a Zeiss 780 confocal microscope. Images were acquired every second (sec) for 4 min. After 15 sec of basal recoding, histamine (100 μM final concentration) was added to the cells. For RhoA2G positive control, cells were treated with Thrombin 1 U/μL. FRET to donor channel ratio at the cell protusion area of individual cell was calculated using image J software.

### Oxygen Consumption Rate (OCR) and ATP measurements

OCRs were measured using a Seahorse Bioscience XF24 Extracellular Flux Analyzer (Seahorse Bioscience, North Billerica, MA) according to the manufacturer’s protocol. Cells were seeded in normal growth medium 24 h before measurement at a density of 5 × 10^4^ cells per well for Hs578t sh Control, sh1 MCU and sh2 MCU cells. The assay was performed in XF minimal basal medium (Seahorse Bioscience) supplemented with 25 mM glucose, 1 mM sodium pyruvate, 4 mM glutamine, 10% FBS at pH 7.4. Cells were pre-incubated in this medium for 1 h before measurement at 37 °C in a humidified atmosphere without CO_2_. Oligomycin A (0.5 μM), FCCP (1 μM), rotenone (0.5 μM), antimycin A (0.5 μM) were used to evaluate mitochondrial respiratory capacity. The data were normalized by the amount of protein present in each well.

Intracellular ATP levels in Hs578t cells expressing sh Control and the two sh MCU RNAs were assessed using ATP Assay Kit (Abcam) following manufacturer’s instructions. Clariostar microplate fluorescence reader (BMG Labtech) was used for the measure of fluorescence (Ex/Em = 535/587 nm).

### BAPTA-AM treatment

BAPTA-AM treatment was performed by incubation of Hs578t cells with 5 μM of BAPTA-AM (Enzo BML CA 411 0025) for 1 h at 37 °C, followed by Calpains activity measurements or immunofluorescence analyses. Cells were tracked for 24 h.

### Ca^2+^ measurements

Cytosolic Ca^2+^ levels were detected in HeLa and Hs578t cells using Fluoforte dye (Enzo Life Sciences). Cells seeded in 96-well plates were incubated with 5 μM Fluoforte for 1 h at 37 °C, in a Ca^2+^-free balanced salt solution (BSS) [121 mM NaCl, 5.4 mM KCl, 0.8 mM MgCl_2_, 6 mM NaHCO_3_, 5.5 mM D-Glucose, 25 mM Hepes (pH 7.3)]. Raw fluorescence values were collected every 0.21 sec for 2 min at 28 °C using Clariostar microplate fluorescence reader (BMG Labtech). After 5 sec of basal line measurement, 100 μM histamine (Sigma) was injected.

For basal Ca^2+^ measurements the Fluoforte fluorescence signals were normalized to total protein levels using Bradford assay.

For ER-Ca^2+^ leakage experiments Fluoforte loaded HeLa cells, transfected with control,or MCU siRNAs, were treated with 10 μM thapsigargin (Enzo Life Sciences) as described previously^55^. Fluorescence signals were recorded using Zeiss 780 confocal microscope. Fluorescence excitation was performed using 488 nm laser and detection filters were set at 533/40 nm using a Zeiss 780 confocal microscope. Images were acquired every sec for 2 min.

SOCE measurements were performed on Hs578t cells expressing sh Control and the two sh-MCU RNAs. Cells were incubated during 1 h with 5 μM Fluoforte dye, and washed with BBS containing 0.1 mM EGTA. After 20 sec of baseline, Thapsigargin 10 μM was added, and 5 min later 2 mM of CaCl_2_ was added.

Mitochondrial Ca^2+^ measurements on siRNA transfected or shRNA expressing Hs578t cells were performed using Rhod-2 AM chemical dye or CEPIA2mt recombinant protein, respectively. Rhod-2 detection was performed as previously described for Fluoforte with the following modifications. Rhod-2 AM dye was incubated at 2.5 μM with 0.2% of pluronic acid (Molecular probes) for 20 min at 37 °C in BBS-Ca^2+^ (BBS supplemented with 2 mM of Ca^2+^), and washed for 20 min with BBS-Ca^2+^at room temperature. Raw fluorescence values were collected every 0.21 sec for 2 min at room temperature using Clariostar microplate fluorescence reader.

For CEPIA2mt detection, Hs578t expressing sh Control or the two sh-MCU RNAs were transfected with pCMV CEPIA2mt (Addgene #58218) and seeded at 40% in 8 well Nunc™ Lab-Tek™ II Chamber Slide. Twenty-four h later, cells were washed with BBS-Ca^2+^. After 10 sec of baseline, 100 μM of histamine was added. Fluorescence excitation was performed using 488 nm laser and detection filters were set at 533/40 nm using a Zeiss 780 confocal microscope. Images were acquired every sec for 5 min.

Mitochondrial Ca^2+^ uptake on isolated mitochondria was performed as previously described^32^ with the following modifications. Hs578t cells were transfected with si1 and si2 MCU siRNAs as well as control siRNA. Forty eight h later, two 10 cm petri dishes were washed 2 times with PBS and cells were scraped with 1 mL ice cold MB buffer (210 mM mannitol, 70 mM sucrose, 1 mM EDTA, 10 mM HEPES [pH 7.5] containing proteases inhibitors). Ca^2+^ measurements were perfomed using Clariostar microplate fluorescence reader (Ex/Em = 492/517 nm).

ER Ca^2+^ filling capacities in Hs578t expressing sh Control and the two sh-MCU RNAs were measured using the ER probe R-CEPIA1er recombinant protein. Cells were transfected with pCMV R-CEPIA1er (Addgene #58216). Cells were seeded at 40% confluence in 8 well Nunc™ Lab-Tek™ II Chamber Slide. Twenty four h later cells were washed with BBS-Ca^2+^ and incubated with ionomycin (10 μM). Fluorescence excitation was performed using 488 nm laser and detection filters were set at 533/40 nm using a Zeiss 780 confocal microscope. Images were acquired every sec for 5 min.

### Detection of Calpains activity and activated Rac1

Calpains activity was measured in MCU and control shRNAs Hs578t cell lines using a Calpains Activity Assay Kit (Abcam #ab65308) following manufacturer’s instructions. Briefly, for every measurement, 1.5 × 10^6^ cells of each condition were lysed with extraction buffer and fluorescence was evaluated using excitation filter: 400 nm ± 15 nm and emission filter: 505 nm ± 15. Activated Rac1 form was detected using RhoA/Rac1/Cdc42 Activation Assay Combo Kit (Cell Biolabs) following the manufacturer’s instructions.

### Western blot analyses and antibodies

Western blots were performed as previously described^56^. The following antibodies were used: MCU (Abcam #Ab121499; dilution: 1/500), F_1_F_0_ ATPase (BD #612518; dilution: 1/1000), Vinculin (Santa Cruz #sc-55465; dilution: 1/2000), Paxillin H114 (Santa Cruz #sc-5574; dilution: 1/1000), Phospho-Paxillin Tyr 118 (Cell Signaling Technology #2541; dilution: 1/1000), Phospho-Myosin light chain 2 (Cell Signaling Technology #3671; dilution: 1/1000), STIM1 (Abcam #Ab57834; dilution: 1/1000) and Rac1 (Cell Biolabs #240106; dilution: 1/1000).

### Immunofluorescence

Hs578t cells were seeded on a glass coverslip in 6-well plates at 50% of confluence. Following cell attachment the medium was removed and cell were fixed with 4% Paraformaldehyde containing 0.3% Triton X100. Cells were washed three times in 1X PBS and incubated with blocking buffer (0.1% Triton X100, 3% BSA in PBS). Vinculin antibody (Sigma #V4505; dilutiom: 1/50) was used to detect the FAPs. Phospho-Myosin light chain-2 antibody (Cell Signaling Technology #3671) was used at 1/50 dilution. F-Actin was stained with rhodamine phalloidin dye (Invitrogen #R415) at 1/200 dilution. Nuclei were visualized using Hoecht 33342 dye (Invitrogen #H3570) at 10 μg/mL. Images were acquired under a Zeiss 780 confocal microscope.

### Statistical analyses

Error bars displayed on graphs represent standard errors of the mean (S.E.M) of at least three independent experiments. Statistical significance was determined using the unpaired two-sample Student’s T-test with the following convention: *P < 0.05, **P < 0.01, ***P < 0.001.

## Additional Information

**How to cite this article**: Prudent, J. *et al.* Mitochondrial Ca^2+^ uptake controls actin cytoskeleton dynamics during cell migration. *Sci. Rep.* **6**, 36570; doi: 10.1038/srep36570 (2016).

**Publisher’s note**: Springer Nature remains neutral with regard to jurisdictional claims in published maps and institutional affiliations.

## Supplementary Material

Supplementary Information
