# Supplementary material for: Mitochondrial Ca2+ uptake controls actin cytoskeleton dynamics during cell migration
Source: Sci Rep. 2016 Nov 9;6:36570. doi: 10.1038/srep36570 (PMC5101530; doi:10.1038/srep36570)
Supplement: Supplementary Information [file srep36570-s1.pdf]

SUPPLEMENTARY INFORMATION

Mitochondrial Ca^{2+} uptake controls cytoskeleton dynamics during cell migration

Authors:

Julien Prudent[1*§], Nikolay Popgeorgiev[2*§], Rudy Gadet[2*], Mathieu Deygas[2], Ruth Rimokh[2] and Germain Gillet[2,3]

Affiliations:

1. Medical Research Council, Mitochondrial Biology Unit, Cambridge Biomedical Campus, Hills Road, Cambridge CB2 0XY, UK.

2. Université de Lyon, Centre de recherche en cancérologie de Lyon, U1052 INSERM, UMR CNRS 5286, Université Lyon I, Hospices civils de Lyon, Centre Léon Bérard, 28 rue Laennec, 69008 Lyon, France.

3. Hospices civils de Lyon, Laboratoire d'anatomie et cytologie pathologiques, Centre Hospitalier Lyon Sud, Chemin du Grand Revoyet, 69495 Pierre Bénite, France

* These authors contributed equally to this work.

§ Corresponding authors: Julien Prudent: julien.prudent@mrc-mbu.cam.ac.uk and Nikolay Popgeorgiev: nikolay.popgeorgiev@univ-lyon1.fr

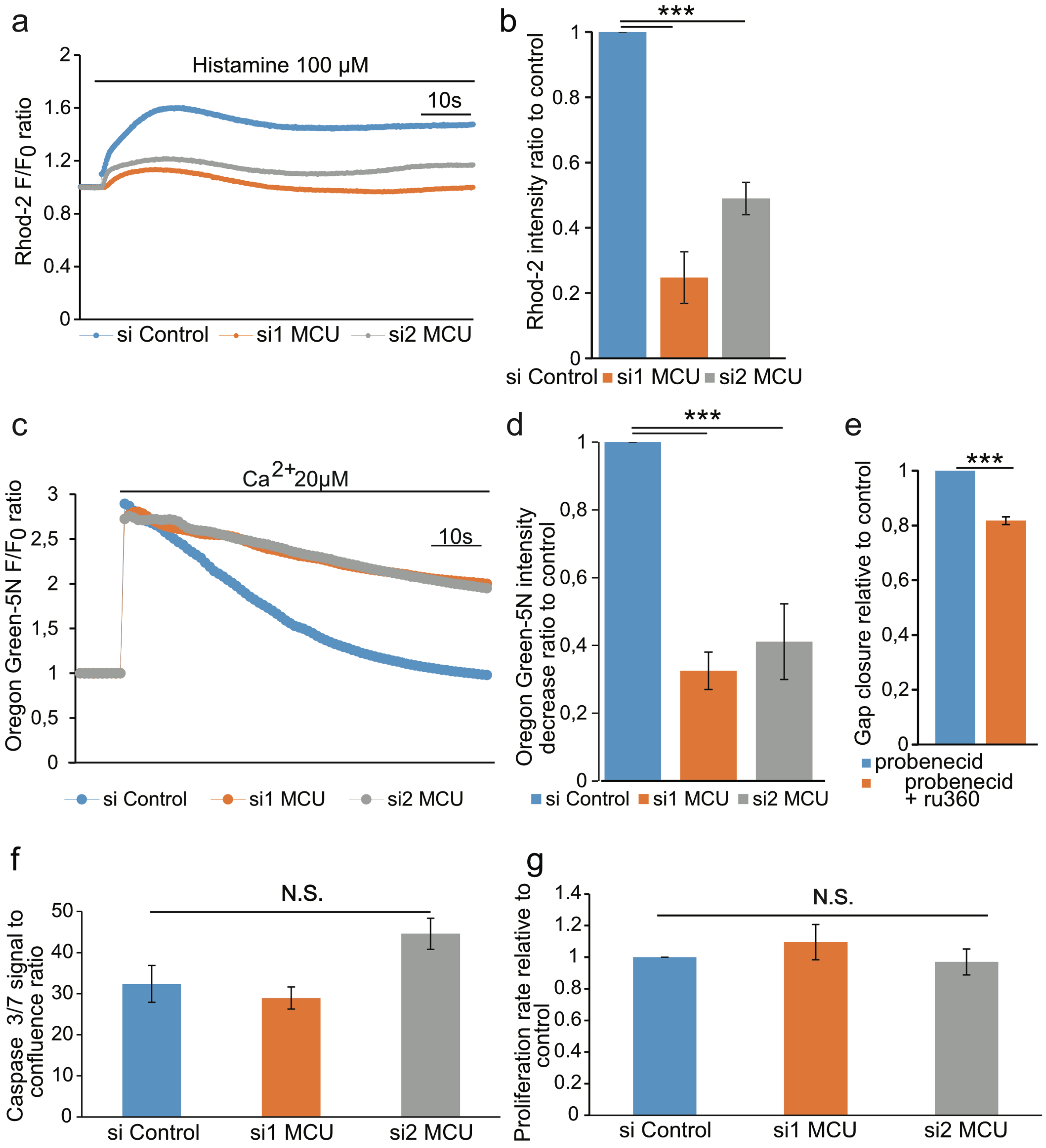

Supplementary Figure S1 related to Figure 1. *Mcu*-silencing in Hs578t cells alters mitochondrial Ca^{2+} uptake, but not apoptosis or cell proliferation.

a. Representative traces of the relative changes in the mitochondrial Rhod-2AM fluorescence intensity normalized to the baseline (F/F_0 ratio) reflecting the mitochondrial Ca^{2+} rise following histamine (100 µM) stimulation in Hs579t cells silenced or not with the 2 *mcu*-siRNA.

b. Histogram depicting the maximum mitochondrial Ca^{2+} increase (F_{max}/F_0 ratio) in Hs578t cells following histamine (100 µM) treatment relative to control, from Fig S1a. (mean ± S.E.M; three independent experiments). ***P < 0.001.

c. Representative traces of the relative changes in the Oregon Green-5N fluorescence intensity normalized to the baseline (F/F_0 ratio), reflecting the mitochondrial Ca^{2+} uptake following calcium (20 µM) stimulation from isolated mitochondria of Hs579t cells silenced or not with the 2 *mcu*-siRNA.

d. Histogram depicting the mitochondrial Ca^{2+} uptake (F_{max}/F_0 ratio) of isolated mitochondria from si-*mcu* treated Hs578t cells following Ca^{2+} (20 µM) boost relative to control, from Fig S1c. (mean ± S.E.M; three independent experiments). ***P < 0.001.

e. Histogram showing the gap closure of Ru360 (60µM) + probenecid (2.5 mM) treated Hs578t cells relative to control (probenecid treated only) (mean ± S.E.M; three independent experiments). ***P < 0.001.

f. Histogram showing the Caspase 3/7 signal to confluence ratio for Hs578t cells silenced with the 2 *mcu*-siRNA (mean ± S.E.M; n=3 independent experiments). N.S.= not significant.

g. Histogram showing the proliferation rate relative to control for Hs578t cells silenced with the 2 *mcu*-siRNA (mean ± S.E.M; n=3 independent experiments). N.S.= not significant; ***P < 0.001.

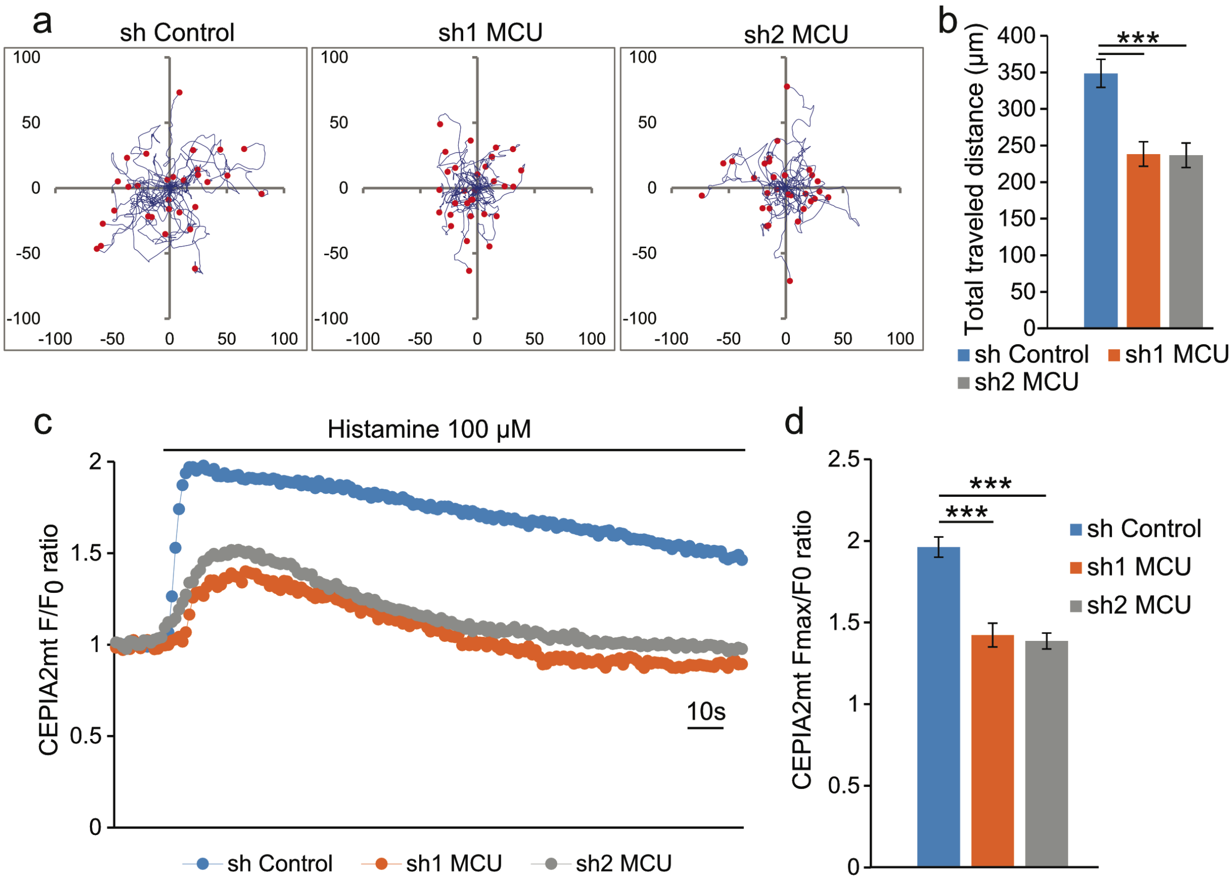

Supplementary Figure S2 related to Figure 2. Stable sh*mcu* silencing in Hs578t cells compromises cell migration as well as mitochondrial Ca^{2+} uptake.

a. Representative single cell tracking experiments highlighting the cell paths (blue line) of isolated Hs578t (red dots) stably expressing sh Control, sh1 or sh2 MCU shRNAs; acquisition time 24 hours.

b. Histogram deduced from the results displayed in Fig. S2a showing the distance of cell migration relative to control (mean ± S.E.M; 30 cells in each experiment; n=3 independent experiments). ***P < 0.001.

c. Representative traces of the relative changes in the fluorescence intensity normalized to the baseline (F/F_0 ratio) reflecting the mitochondrial Ca^{2+} rise following histamine (100 µM) stimulation. The mitochondrial Ca^{2+} increase was detected using the mitochondrial localized CEPIA2mt Ca^{2+} sensor transiently expressed in Hs578t cells. *Mcu* knockdown using specific

shRNA (sh1 and sh2) significantly decreased the mitochondrial calcium uptake compared to control cells transfected with negative control shRNA (sh Control).

d. Histogram depicting the maximum mitochondrial Ca^{2+} increase (F_{max}/F_0 ration) in Hs578t cells following histamine (100 μM) treatment, from Fig S2c (mean ± S.E.M; n=3 independent experiments). ***P < 0.001.

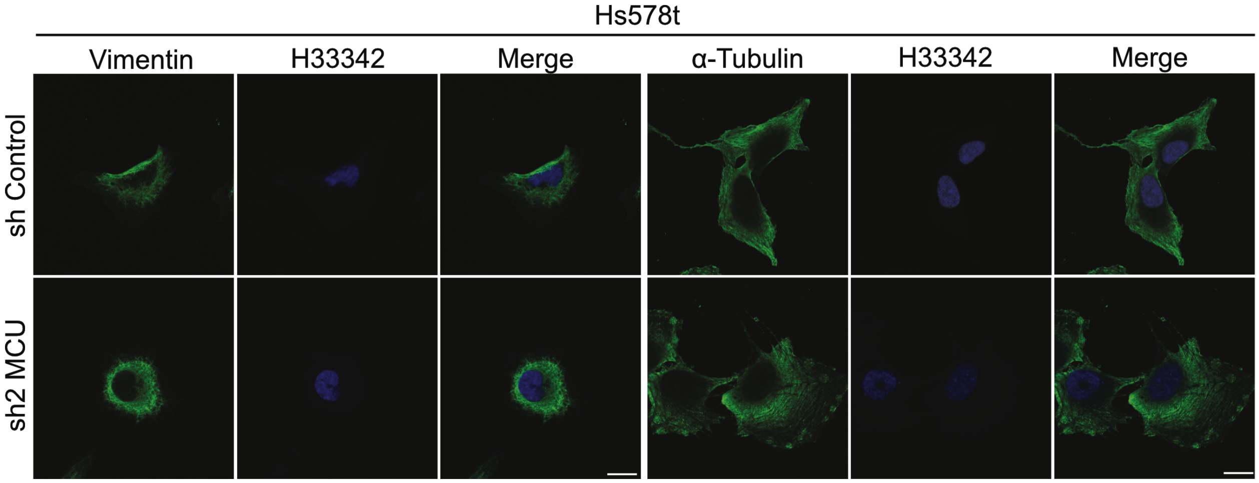

Supplementary Figure S3 related to Figure 3. Mitochondrial Ca^{2+} uptake impairment does not lead to microtubules and intermediate filaments modifications.

Representative confocal images of sh Control and sh2 MCU Hs578t cells stained for intermediate filaments and microtubules using anti-vimentin and anti-tubulin antibodies, respectively. Intermediate filaments and microtubules organization was not affected in the *mcu*-silenced cells compared to controls. Cells were stained additionally with Hoechst 33342 (blue) dye. Scale bar: 20 μm.

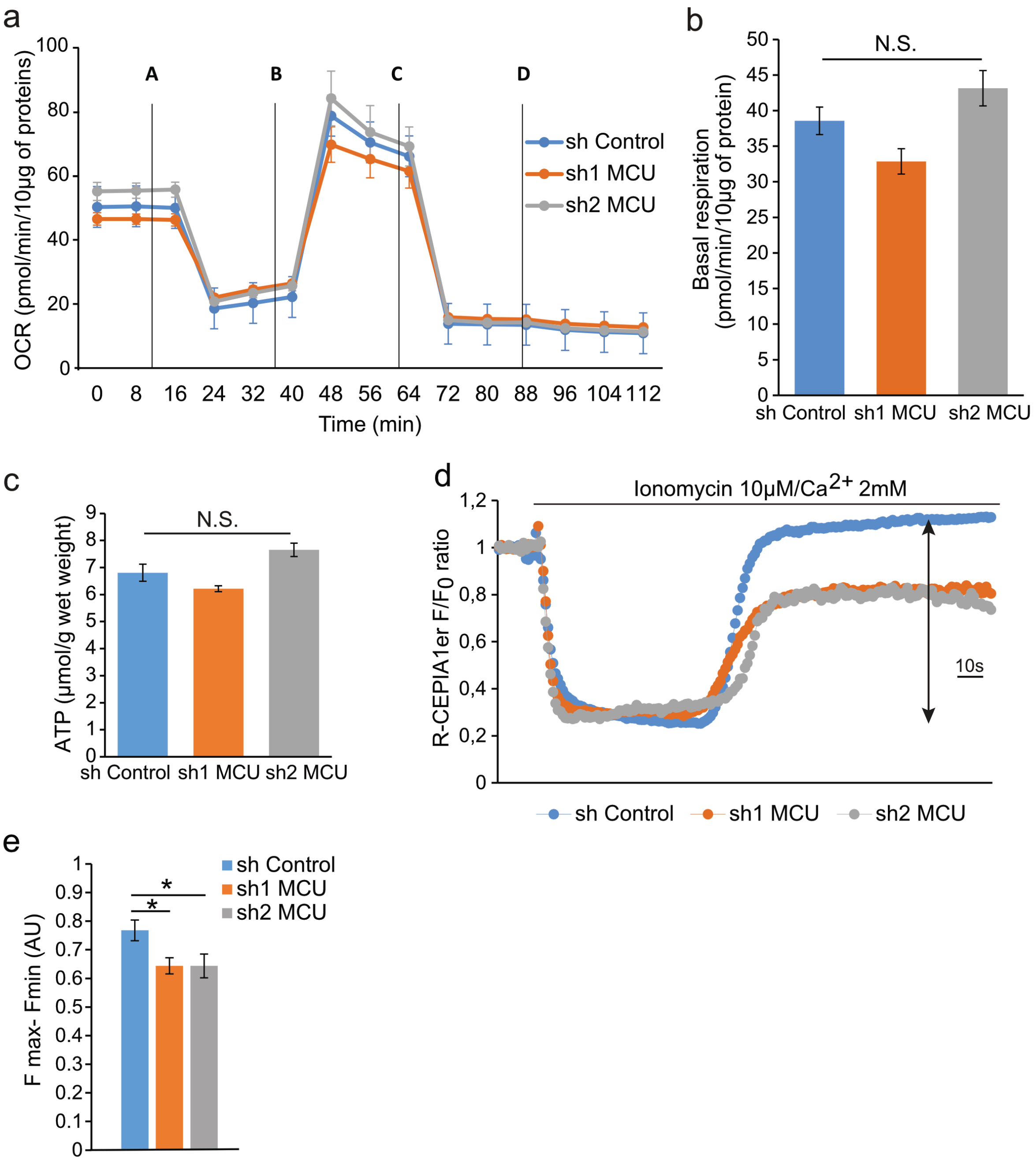

Supplementary Figure S4 related to Figure 4. Mitochondrial Ca^{2+} uptake impairment leads to SOCE default but not to ATP production loss.

a. Raw data of Oxygen Consumption Rate (OCR) determined by the SeaHorse XF24 analyser. OCR was measured continuously in Hs578t expressing sh Control, sh1 MCU or sh2 MCU shRNAs throughout the experimental period at baseline followed by the addition of the indicated

drugs. A: Oligomycin (0.5 μM), B: FCCP (1 μM), C: Rotenone (0.5 μM), D: Antimycin A (0.5 μM). OCR was normalized to protein level. (mean ± S.E.M; n=3 independent experiments).

b. Histogram depicting basal respiration calculated from the first baseline mean (pmol/min/10μg protein) in Hs578t cells stably expressing sh Control, sh1 MCU or sh2 MCU shRNAs (mean ± S.E.M; n=3 independent experiments). N.S.= not significant.

c. Total ATP levels in Hs578t cells stably expressing sh Control, sh1 MCU or sh2 MCU shRNAs. (mean ± S.E.M; n=3 independent experiments). N.S.= not significant.

d. Representative traces of the relative changes of the ER targeted R-CEPIA1er fluorescence intensities normalized to the baseline (F/F_0 ratio). Ca^{2+} ionophore, ionomycin (10 μM), stimulation in the presence of 2 mM Ca^{2+} induces ER Ca^{2+}-depletion followed by rapid ER filling through SOCE. Compared to control cells, sh1 and sh2 MCU expressing cells possess lower ER filling capacities (Fmax-Fmin difference, black arrow).

e. Histogram depicting the ER filling capacities (Fmax-Fmin difference) of Hs578t cells stably expressing sh Control, sh1 MCU or sh2 MCU shRNAs. (mean ± S.E.M; n=3 independent experiments). $^*P < 0.05$.

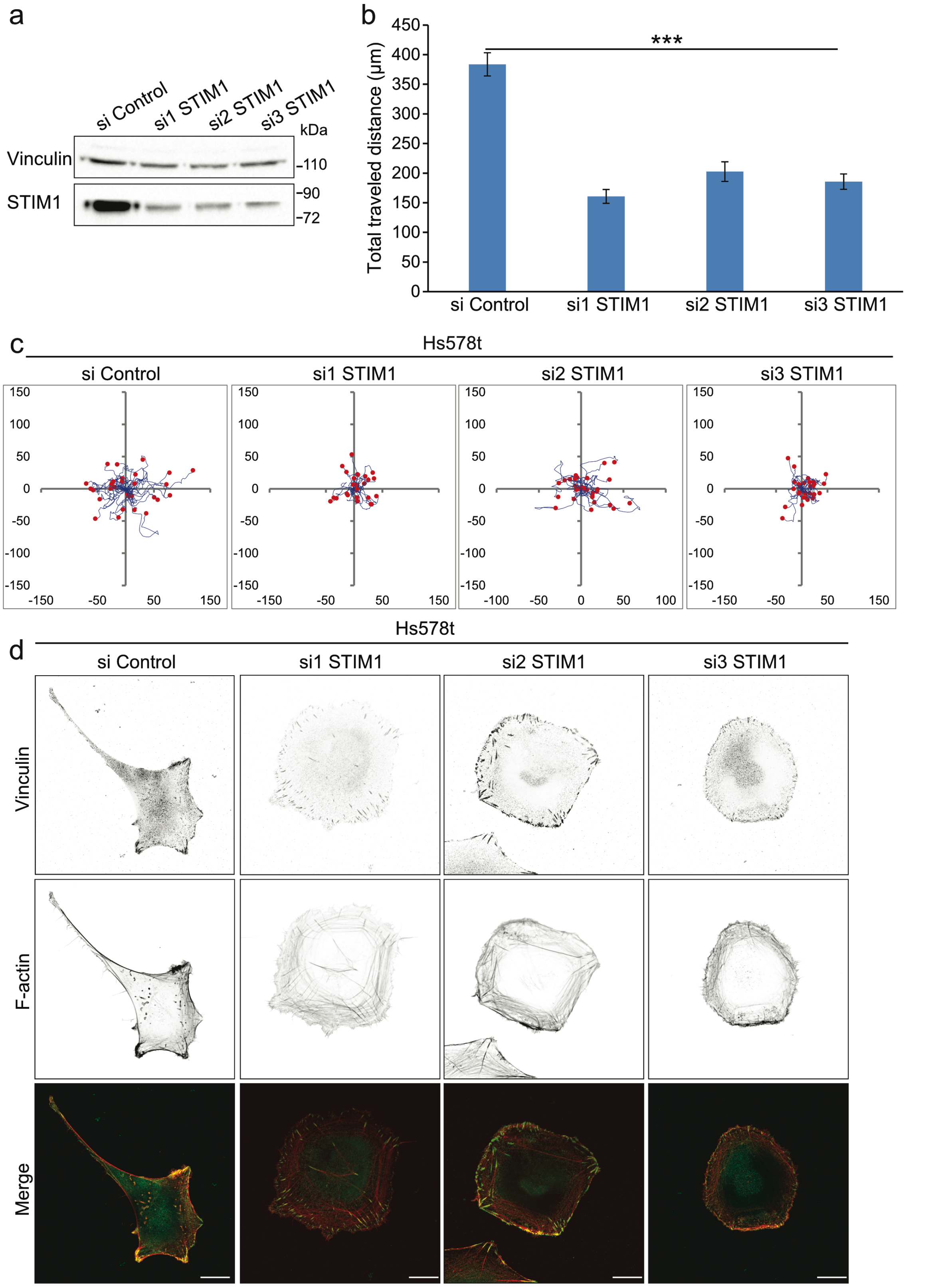

Supplementary Figure S5 related to Figure 5. STIM1 silencing leads to cell migration and cell polarization defects.

a. Immunoblot blot showing the effect of three siRNAs (si1, si2 and si3) on STIM1 expression in Hs578t cells. Scrambled siRNA (si Control) was used as negative control. Vinculin was used as loading control.

b. Histogram representing the distance of cell migration of *stim1*-silenced cells relative to control (mean ± S.E.M; 30 cells in each experiment; n=3 independent experiments). ***P<0.001.

c. Representative single cell tracking experiments highlighting the cell paths (blue lines) of isolated Hs578t cells (red dots), silenced or not for STIM; acquisition time 24 hours.

d. Confocal images of si control and si1, si2 or si3 STIM1 stained for Vinculin and F-actin using anti-Vinculin antibody and phalloidine rhodamine probe, respectively. Images are presented as negative LUT mask. Scale bar: 20µm.
